# Diagnostic Value of Serum Lactate Dehydrogenase Level Measured in the Emergency Department in Predicting Clinical Outcome in Out-of-Hospital Cardiac Arrest: A Multicenter, Observational Study

**DOI:** 10.3390/jcm12083006

**Published:** 2023-04-20

**Authors:** Jihyun Kim, Yong Won Kim, Tae-Youn Kim

**Affiliations:** Department of Emergency Medicine, Dongguk University Ilsan Hospital, Dongguk University College of Medicine, Goyang 10326, Republic of Korea; wusl953@gmail.com (J.K.); chiefong@naver.com (Y.W.K.)

**Keywords:** biomarker, cardiopulmonary resuscitation, lactate dehydrogenase, outcome assessment

## Abstract

Introduction: Out-of-hospital cardiac arrest (OHCA) is complex, and risk stratification tools have the potential to include components other than clinical risk indicators, thus requiring extensive studies. Simple and accurate biomarkers for OHCA patients with poor prognoses are still needed. Serum lactate dehydrogenase (LDH) has been identified as a risk factor in patients with various diseases, such as cancer, liver disease, severe infections, and sepsis. The primary aim of this study was to assess the accuracy of LDH values at initial presentation in the emergency department (ED) in predicting the clinical outcome in OHCA. Methods: This retrospective multicenter observational study was performed in the ED of two tertiary university hospitals and one general hospital between January 2015 and December 2021. All patients with OHCA who visited the ED were included. The primary outcome was the sustained return of spontaneous circulation (ROSC; >20 min) after advanced cardiac life support (ACLS). The secondary outcome was survival to discharge (including home care and nursing care discharge) among patients with ROSC. The neurological prognosis was considered a tertiary outcome in patients who survived to discharge. Results: In total, 759 patients were enrolled in the final analysis. The median LDH level in the ROSC group was 448 U/L (range: 112–4500), which was significantly lower than that in the no-ROSC group (*p* < 0.001). The median LDH level in the survival-to-discharge group was 376 U/L (range: 171–1620), which was significantly lower than that in the death group (*p* < 0.001). Using the adjusted model, the odds ratio of the LDH value (≤634 U/L) for primary outcomes was 2.418 (1.665–3.513) and the odds ratio of LDH value (≤553 U/L) for secondary outcomes was 4.961 (2.184–11.269). Conclusions: In conclusion, the serum LDH levels of patients with OHCA measured in the ED can potentially serve as a predictive marker for clinical outcomes such as ROSC and survival to discharge, although it may be difficult to predict neurological outcomes.

## 1. Introduction

Out-of-hospital cardiac arrest (OHCA) results in 185,000–450,000 deaths each year, representing 7–18% of all deaths in the United States [1]. The clinical outcomes of OHCA patients are influenced by various factors, including early detection, basic life support, duration of cardiopulmonary resuscitation, and initial presenting shockable rhythm [2,3]. The cardiopulmonary resuscitation (CPR) guidelines recommend a multidisciplinary evaluation to determine prognosis, mainly because the single most accurate prognostic factor has not yet been verified [4,5]. OHCA patients require advanced care such as intensive care and targeted temperature management compared to other disease groups, and this advanced health care increases the socio-economic burden associated with the length of stay, medical procedures, and treatment systems [6]. Determining the severity earlier in the course of OHCA would enable the administration of critical care to reduce medical resources [7]. Laboratory studies during advanced cardiovascular life support (ACLS) are conducted to determine the reversible cause of cardiac arrest in patients, and a serial follow-up of biomarkers performed at this time is used for prognosis, such as survival to discharge and neurologic outcome [8,9]. Serum biomarkers such as troponin and B-type natriuretic peptide have been studied as potential early predictors of OHCA [10,11], although these biomarkers have low accuracy when used alone and have been recommended to be used in combination [12,13]. OHCA is complex, and risk stratification tools have the potential to include components other than clinical risk indicators, which requires extensive studies [14]. Simple and accurate biomarkers for OHCA patients with poor prognoses are still needed. It would be desirable to find a convenient and cost-effective serum marker that could ideally predict the outcome of OHCA in the emergency department (ED).

Serum lactate dehydrogenase (LDH) has been identified as a risk factor in patients with various diseases, such as cancer, liver disease, severe infections, and sepsis [15]. Very high outlier values of serum LDH measured in all patients, regardless of symptom onset, hospitalization or outpatient, were found to be more likely to be associated with 72-h mortality [16]. Elevated serum LDH during hospitalization was positively related with in-hospital mortality in patients with acute aortic dissection [17]. In patients with aneurysmal subarachnoid hemorrhage, the LDH level is an independent predictor of all-cause mortality [18]. High serum LDH levels predicted unfavorable clinical outcomes after intravenous thrombolysis in acute ischemic stroke patients [19]. The LDH enzyme that catalyzes the reversible conversion of pyruvate to lactate is known to be a marker of tissue damage [20]. The LDH test is advantageous because it can be quickly performed in an ED [21]. 

Currently, no information is available on the clinical usefulness of serum LDH in the ED with respect to the prediction of clinical outcomes in OHCA in the early phase. Therefore, the primary aim of this study was to assess the accuracy of LDH values at initial presentation in the ED in predicting the clinical outcome in OHCA. We hypothesized that LDH levels might be elevated in OHCA patients in the ED, along with a high risk of poor clinical outcome.

## 2. Materials and Methods

### 2.1. Study Design and Setting

This retrospective multicenter observational study was performed in the ED of two tertiary university hospitals and one general hospital between January 2015 and December 2021. This study was approved by the Institutional Review Board (IRB) of Wonju Severance Christian Hospital (IRB No. CR322004), Dongguk University Ilsan Hospital, Dongguk University (IRB No. DUIH 2022-02-027), and that of National Health Insurance Service Ilsan Hospital (IRB No. NHIMC 2022-03-030-001). The study protocol conformed to the ethical guidelines of the Declaration of Helsinki (1975) and its amendments. As the study involved retrospective and observational analyses, the requirement for informed consent was waived, and patient records and information were anonymized before analysis. Emergency medical technicians provide both basic and advanced life support for a minimum of 5 min at the scene. If the return of spontaneous circulation (ROSC) cannot be achieved, the patient is transported to the nearest ED. After ROSC is achieved, the patient is referred to the nearest hospital to receive post-cardiac arrest care, including targeted temperature management.

### 2.2. Participants

From January 2015 to December 2021, all patients with OHCA who visited the ED were included. The total number of enrolled patients was 2375. All patients underwent treatment in accordance with the current ACLS guidelines. The exclusion criteria were as follows: (1) aged < 18 years, (2) traumatic arrest, (3) missing LDH values, and (4) death on arrival or patients with do-not-resuscitate orders.

### 2.3. Study Variables

The following epidemiologic and biomarker parameters were obtained from the medical records retrospectively: age, sex, the witness of cardiac arrest, bystander cardiopulmonary resuscitation (CPR), initial shockable rhythm, total CPR time, in-hospital CPR time, total epinephrine dose, and LDH level at the ED. LDH was measured within 2 to 3 cycles of CPR during in-hospital ACLS.

### 2.4. Study Endpoints

The primary outcome was sustained ROSC (>20 min) after ACLS. Secondary outcomes were divided into two patient groups: death after ROSC (including death in the ED or intensive care unit) and survival to discharge (including home care and nursing care discharge) among patients with ROSC. However, patients were excluded when they were referred to other institutions in ED or could not receive post-cardiac arrest care because they were transferred to other institutions within the ICU.

The neurological prognosis was considered a tertiary outcome in patients who survived to discharge and was graded using Cerebral Performance Categories (CPCs), which was measured at the time of hospital discharge and assessed for outcomes. Favorable neurological outcomes were defined as CPC 1–2 and poor neurological outcomes were defined as CPC 3–5.

### 2.5. Statistical Analysis

Continuous data are presented as means with standard deviations or medians (interquartile ranges), followed by normality tests. Normally distributed data were assessed using the Shapiro–Wilk test. Categorical variables are presented as counts and percentages. Continuous data were analyzed using Student’s *t*- or Mann–Whitney *U* test, as appropriate. Categorical data were analyzed using the chi-squared test or Fisher’s exact test, as appropriate. To calculate the effect size, Cramer’s v and eta squared coefficients were calculated. To evaluate the factors contributing to clinical outcomes, including ROSC, survival to discharge, and favorable neurologic outcomes, univariable and multivariable logistic regression analyses were performed, which are presented with odds ratios (OR) and 95% confidence intervals (CI). Variables with a *p*-value < 0.2 in univariable logistic regression analysis were included in the multivariable logistic regression analysis. A restricted cubic spline curve was fitted to visualize differences in the OR of ROSC, survival to discharge, and neurologic outcome according to LDH. *p*-values of <0.05 were considered statistically significant, all analyses were performed using SPSS ver. 23 (IBM Corp., New York, NY, USA) and R statistical software (version 3.6.3; R Foundation for Statistical Computing, Vienna, Austria).

## 3. Results

### 3.1. Baseline Characteristics

**Figure 1:**
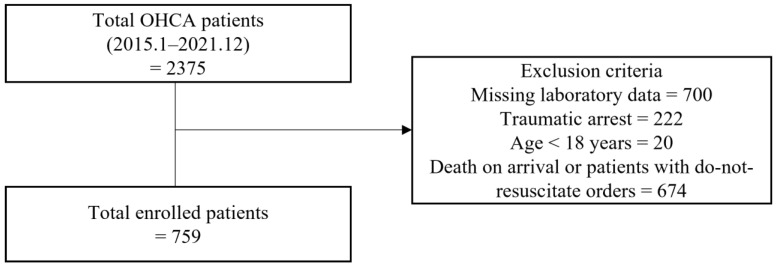
Flowchart of patient screening and selection during the study enrollment process. OHCA, out-of-hospital cardiac arrest.

During the study period, 2375 patients with OHCA were admitted to the ED. Among them, 1616 patients were excluded from the analysis because of the following factors: absence of laboratory biomarker data (*n* = 700), trauma (*n* = 222), age < 18 years (*n* = 20), death on arrival or those with do-not-resuscitate orders (*n* = 674). Therefore, 759 patients were enrolled in the final analysis (Figure 1). Among the patients with ROSC, those who were transferred were excluded (*n* = 34).

Among 759 patients, 328 had ROSC, of whom 64 survived to discharge. Among the patients with survival to discharge, 27 had favorable neurologic outcomes. The ROSC group was relatively younger, and the initial presenting rhythm of the ROSC group was a more frequent shockable rhythm (*p* = 0.001 and 0.009, respectively). The total CPR time was lower in the ROSC group than in the no-ROSC group (*p* = 0.000), and the total administered epinephrine dose was lower in the ROSC group (*p* = 0.000). The median LDH level in the ROSC group was 448 U/L (range: 112–4500), which was significantly lower than that in the no-ROSC group (623 U/L; range: 117–4500) (Table 1). Of the 328 patients with ROSC, 34 were excluded from the analysis because they were transferred and their survival to discharge was unknown; 64 patients survived to discharge. The survival-to-discharge group was relatively younger and had a more frequent initial shockable rhythm (*p* < 0.001 and 0.002, respectively). The total CPR time and total administered epinephrine doses were lower in the survival-to-discharge group (*p* = 0.001 and 0.005, respectively). The median LDH level in the survival-to-discharge group was 376 U/L (range: 171–1620), which was significantly lower than that in the death group (486 U/L; range: 112–4500) (Table 1). Of the 64 patients with survival to discharge, 27 had favorable neurologic outcomes. The favorable neurologic outcome group was relatively younger and had a more frequent shockable rhythm (*p* = 0.003 and 0.001, respectively). The out-of-hospital CPR time and total CPR time were lower in the favorable neurologic outcome group (*p* = 0.001 and 0.016, respectively). The median LDH level in the favorable neurologic outcome was 354 U/L (range: 171–1620), which was not significantly different from that in the poor neurologic outcome group (388 U/L; range: 211–829) (Table 1). Calculated effect size coefficients by clinical outcomes are shown in Supplementary Table S1.

### 3.2. Diagnostic Value of LDH in the Clinical Outcomes of OHCA 

**Figure 2:**
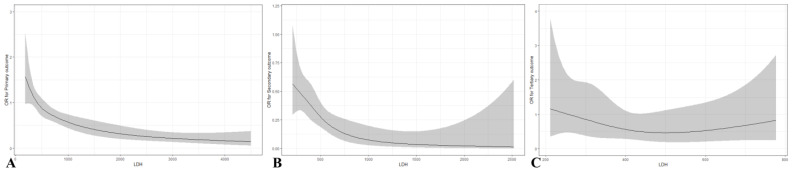
The trend of odds ratio of outcomes according to LDH level. (**A**) Primary outcome (ROSC); (**B**) Secondary outcome (Survival discharge); and (**C**) Tertiary outcome (Favorable neurologic outcome).

ROC curves were used to assess the predictive ability of LDH for clinical outcomes in OHCA patients (Supplementary Figure S1). The optimum cut-off values for LDH were as follows: for primary outcomes ≤634 U/L (sensitivity 74.4%, specificity 48.7%); for secondary outcomes ≤553 U/L (sensitivity 87.5%, specificity 42.6%); and for tertiary outcomes ≤483 U/L (sensitivity 81.5%, specificity 37.8%) (Supplementary Figure S1). A cubic spline curve was fitted to visualize the difference in the OR of clinical outcomes according to the LDH level. The OR of primary and secondary outcomes decreased proportionally to the LDH level, whereas the OR of tertiary outcomes was not correlated with the LDH level (Figure 2).

### 3.3. Predictive Value of LDH in the Clinical Outcomes of OHCA

Logistic regression analysis was performed to assess the correlation between LDH and clinical outcomes of out-of-hospital cardiac arrest. The probabilities of primary and secondary outcomes increased significantly when the LDH level was below the respective cut-off value. However, the probability of tertiary outcome was not significantly associated with LDH. Using the adjusted model, including age, witness, initial shockable rhythm, in-hospital CPR time, out-of-hospital CPR time, total CPR time, and total epinephrine dose, the OR of LDH (≤634 U/L) for primary outcomes was 2.418 (1.665–3.513); the OR of LDH (≤553 U/L) for secondary outcomes was 4.961 (2.184–11.269); and the OR of LDH (≤483 U/L) for tertiary outcomes was 3.192 (0.691–14.743) (Table 2).

## 4. Discussion

In this study, we verified LDH measured in the ED as an independent predictive factor associated with the clinical outcome of patients with cardiac arrest, and suggested a cut-off value for each clinical outcome. In this cohort, the group of patients with serum LDH ≤ 634 U/L measured in the ED was an independent factor associated with ROSC, with an adjusted OR of 2.418 (95% CI, 1.665–3.513). Among patients with ROSC, serum LDH ≤ 553 U/L was an independent factor for survival to discharge, with an adjusted OR of 4.961 (2.184–11.269). However, in the multivariate logistic analysis of favorable neurological outcome in patients who survived to discharge, serum LDH in the ED was not an independent variable, with an adjusted OR of 3.192 (95% CI, 0.691–14.743). According to the cubic spline curve, the OR trend for the primary and secondary outcomes decreased proportionally with serum LDH in the ED. The key difference between previous studies and the current study is that this study was conducted in the ED, whereas previous studies were conducted in the intensive care unit. This study aimed to determine the clinical implication of LDH level that was measured during ACLS in terms of predicting clinical outcomes in patients with OHCA. In this cohort, LDH was confirmed to be an independent predictor of ROSC and survival to discharge. However, its accuracy is difficult to use as a single parameter, and it has been identified as a factor that is difficult to use, especially for neurologic outcomes. 

As in previous studies, it may be helpful to provide accurate information through integrated hematological evaluation of the patient’s expected clinical outcome rather than a single biomarker [10]. The onset of OHCA itself may lead to elevated serum LDH levels [22]. Lactate dehydrogenase (LDH) enzyme is found in several tissues of the body, including the heart. Elevated levels of LDH in the blood have been associated with various cardiac conditions, including acute myocardial infarction and heart failure [23]. During an OHCA event, as the heart muscle cells become damaged due to lack of oxygen, the LDH enzyme is released into the bloodstream, leading to elevated serum levels of LDH [24]. Elevated serum LDH levels indicate the severity of the myocardial injury, which can be a prognostic marker for clinical outcomes after resuscitation. Previous studies have investigated the association between LDH levels and clinical outcomes in patients with cardiac arrest [15,22,25]. Higher levels of LDH measured in the intensive care unit were associated with increased mortality in 374 cardiac arrest patients, according to a previous study [15]. A study evaluating the use of cerebrospinal fluid LDH levels as a prognostic marker for neurological outcomes in patients with cardiac arrest found that cerebrospinal fluid LDH levels measured 48 h after resuscitation were significantly higher in patients who had poor neurological outcomes at 3 months than in those who had good neurological outcomes [25]. Another study evaluated serum LDH at 48 h in the intensive care unit and predicted poor neurological outcomes, with good prognostic values at 48 h and 72 h. This study focused on 95 comatose OHCA patients who were treated using targeted temperature management [22]. 

According to the CPR guidelines, a multimodal approach is recommended for determining the clinical prognosis. Biomarkers are more stable and allow for objective patient assessment [26]. Procalcitonin was proposed as an accurate predictor of poor outcomes in OHCA patients receiving targeted temperature management, as it is related to hypoperfusion and activation of inflammatory pathways due to cardiac arrest [27]. NT-proBNP serum levels are increased in comatose OHCA patients and are independently associated with poor neurological outcomes [28]. However, rather than these single parameters, a combination of blood markers and clinical parameters may help improve prognostic assessment and early decision-making in patients with cardiac arrest [10]. 

In determining the prognosis of cardiac arrest, various scoring systems have been designed for different stages of cardiac arrest [29] and for each stage of sudden cardiac arrest. A typical scoring system for predicting ROSC is the ROSC after cardiac arrest (RACA) score, which consists of sex, age, etiology, the witness of arrest, location of arrest, initial shockable rhythm, bystander CPR, and emergency medical system arrival time. As there are many variables, it is difficult to perform immediate management [30]. The cardiac arrest hospital prognosis (CAHP) score was designed for patients admitted to an intensive care unit and is used to predict poor neurologic outcomes [31]. The CAHP score consists of non-shockable rhythm, arterial pH, age, arrest setting, no-flow time, low-flow time, and dose of epinephrine given during the arrest; therefore, it is composed of many variables like the RACA score. Through this study, we suggest that serum LDH measured in the ED can be used as a potential predictive marker for clinical outcomes of ROSC and survival to discharge. The possibility that LDH can also be grafted to various scoring systems in further studies is presented.

This study had a few limitations. First, this is a retrospective study with many excluded patients. Second, the time of LDH measurement was different in all patients. All LDH levels were measured within the ACLS of the patient visiting the ED. However, caution is advised when interpreting our results because the durations of OHCA and CPR were different. Third, unlike most previous studies, no follow-up data on LDH levels were included in our study. Further follow-up studies are needed to properly establish the values indicated in our results. Fourth, as a multicenter study, a limitation of this study is that 700 OHCA patients were not enrolled because serum LDH levels could not be measured at the initial visit in all cases. This might have caused a selection bias, which could also limit the generalizability of our study. Despite these limitations, the measurement of serum LDH levels in the emergency room has the potential to be a valuable predictive tool for clinical outcomes in patients with OHCA.

## 5. Conclusions

In conclusion, serum LDH levels of patients with OHCA measured in the ED are associated with ROSC and survival to discharge, but not with favorable neurologic outcome and therefore cannot be used to predict neurologic outcome.

## Figures and Tables

**Table 1:** Baseline characteristics of patients.

	Primary Outcome			Secondary Outcome			Tertiary Outcome		
	No-ROSC (*n* = 431)	ROSC (*n* = 328)	*p*-Value	Death (*n* = 230)	Survival to Discharge (*n* = 64)	*p*-Value	Poor (*n* = 37)	Favorable (*n* = 27)	*p*-Value
Age (years) ^a^	70.49 ± 15.55	66.88 ± 15.73	0.001	67.96 ± 15.30	58.83 ± 15.62	0.000	63.46 ± 16.56	52.48 ± 11.80	0.003
Male sex, *n* (%) ^b^	265 (61.5%)	191 (58.2%)	0.406	132 (57.4%)	42 (65.6%)	0.298	25 (67.6%)	17 (63.0%)	0.907
Hypertension, *n* (%) ^b^	197 (45.7%)	140 (42.7%)	0.449	99 (43.0%)	26 (40.6%)	0.839	18 (48.6%)	8 (29.6%)	0.203
Diabetes mellitus, *n* (%) ^b^	120 (27.8%)	102 (31.1%)	0.370	68 (29.6%)	21 (32.8%)	0.729	14 (37.8%)	7 (25.9%)	0.464
Bystander CPR, *n* (%) ^b^	227 (52.7%)	191 (58.2%)	0.146	129 (56.1%)	39 (60.9%)	0.582	21 (56.8%)	18 (66.7%)	0.587
Witnessed, *n* (%) ^b^	206 (47.8%)	197 (60.1%)	0.001	134 (58.3%)	41 (64.1%)	0.489	22 (59.5%)	19 (70.4%)	0.526
Initial shockable rhythm, *n* (%) ^b^	27 (6.3%)	39 (11.9%)	0.009	22 (9.6%)	16 (25.0%)	0.002	3 (8.1%)	13 (48.1%)	0.001
Out-of-hospital CPR time ^a^	27 (0–174)	21 (1–75)	<0.001	22 (1–75)	13.5 (1–68)	0.010	20 (1–52)	4 (1–68)	0.001
In-hospital CPR time ^a^	24 (0–179)	9(0–102)	<0.001	10.5 (1–102)	7 (0–35)	0.002	7 (2–35)	7 (0–33)	0.728
Total CPR time (min) ^a^	52 (3–194)	31 (2–134)	<0.001	32 (2–134)	25.5 (2–72)	0.001	29 (3–67)	13 (2–72)	0.016
Total epinephrine dose (mg) ^a^	8 (0–60)	3 (0–34)	<0.001	4 (1–34)	3 (0–12)	0.005	3 (1–12)	3 (0–11)	0.660
LDH (U/L), reference value (<290 U/L) ^a^	623 (112–4500)	448 (117–4500)	<0.001	486 (112–4500)	376 (171–1620)	<0.001	388 (211–829)	354 (171–1620)	0.362

Abbreviations: ROSC, return of spontaneous circulation; CPR, cardiopulmonary resuscitation; LDH, lactate dehydrogenase. ^a^ Mann–Whitney *U* test. ^b^ Chi-Square Tests, Continuity Correction.

**Table 2:** Association of LDH level with clinical outcomes.

	Primary Outcome (ROSC)		Secondary Outcome		Tertiary Outcome	
	Univariable (OR, 95% CI)	Multivariable (OR, 95% CI)	Univariable (OR, 95% CI)	Multivariable (OR, 95% CI)	Univariable (OR, 95% CI)	Multivariable (OR, 95% CI)
Age (years)	0.985 (0.976–0.995)	0.971 (0.959–0.982)	0.965 (0.948–0.982)	0.968 (0.948–0.988)	0.950 (0.915–0.987)	0.961 (0.918–1.005)
Male sex	1.145 (0.854–1.535)	-	1.417 (0.795–2.527)	-	1.225 (0.433–3.471)	-
Hypertension	0.885 (0.662–1.181)	-	0.905 (0.516–1.590)	-	0.444 (0.156–1.267)	-
Diabetes mellitus	1.170 (0.854–1.602)	-	1.163 (0.643–2.107)	-	0.575 (0.194–1.706)	-
Bystander CPR	1.253 (0.938–1.674)	-	1.221 (0.694–2.150)	-	1.524 (0.543–4.273)	-
Witnessed	1.643 (1.228–2.197)	1.496 (1.045–2.141)	1.277 (0.719–2.267)	-	1.619 (0.564–4.651)	-
Initial shockable rhythm	2.019 (1.208–3.374)	1.501 (0.771–2.925)	3.152 (1.540–6.451)	2.749 (1.173–6.443)	10.524 (2.592–42.727)	9.826 (1.807–53.438)
Out-of-hospital CPR time	0.965 (0.955–0.975)	1.076 (0.874–1.325)	0.975 (0.956–0.996)	1.301 (0.509–3.327)	0.944 (0.905–0.986)	0.934 (0.854–1.023)
In-hospital CPR time	0.906 (0.890–0.921)	0.789 (0.596–1.045)	0.955 (0.922–0.989)	1.156 (0.431–3.098)	1.014 (0.955–1.076)	-
Total CPR time	0.947 (0.938–0.956)	0.901 (0.732–1.109)	0.972 (0.955–0.988)	0.753 (0.295–1.922)	0.963 (0.930–0.997)	0.997 (0.928–1.072)
Total epinephrine dose (mg)	0.748 (0.711–0.788)	2.049 (1.195–3.515)	0.875 (0.789–0.971)	1.350 (0.536–3.398)	1.021 (0.853–1.223)	-
LDH (U/L), cut-off	2.760 (2.021–3.770) ^a^	2.418 (1.665–3.513) ^a^	5.197 (2.369–11.400) ^b^	4.961(2.184–11.269) ^b^	2.678 (0.826–8.686) ^c^	3.192 (0.691–14.743) ^c^

Abbreviations: ROSC, return of spontaneous circulation; OR, odds ratio; CI, confidence interval. ^a^ LDH ≤ 634 U/L. ^b^ LDH ≤ 553 U/L. ^c^ LDH ≤ 483 U/L.

## Data Availability

Data cannot be shared publicly because of the consent of personal information. The data can be accessed with permission from the corresponding author whose contact information is as follows: suffo41@naver.com.

## Supplementary Materials

The following supporting information can be downloaded at: https://www.mdpi.com/article/10.3390/jcm12083006/s1, Figure S1. (A) Primary outcome (ROSC). (B) Secondary outcome (Survival discharge). (C) Tertiary outcome (Favorable neurologic outcome); Table S1. Effect size coefficients by clinical outcomes.
