# Supplementary material for: Diagnostic Value of Serum Lactate Dehydrogenase Level Measured in the Emergency Department in Predicting Clinical Outcome in Out-of-Hospital Cardiac Arrest: A Multicenter, Observational Study
Source: J Clin Med. 2023 Apr 20;12(8):3006. doi: 10.3390/jcm12083006 (PMC10146741; doi:10.3390/jcm12083006)
Supplement: Supplementary file 1 [file jcm-12-03006-s001.zip › jcm-2337972-supplementary.pdf]

Supplementary Materials

Table S1. Effect size coefficients by clinical outcomes.

	**Primary Outcome (*n* = 759)**	**Secondary Outcome (*n* = 294)**	**Tertiary Outcome (*n* = 64)**
	**Effect Size**	**Effect Size**	**Effect Size**
Age (years)[a]	0.013	0.061	0.137
Male sex, *n* (%) [b]	0.033	0.069	0.048
Hypertension, *n* (%) [b]	0.030	0.020	0.191
Diabetes mellitus, *n* (%) [b]	0.035	0.029	0.125
Bystander CPR, *n* (%) [b]	0.055	0.040	0.100
Witnessed, *n* (%) [b]	0.122	0.049	0.112
Initial shockable rhythm, *n* (%) [b]	0.099	0.190	0.457
Out-of-hospital CPR time [a]	0.069	0.022	0.171
In-hospital CPR time [a]	0.255	0.031	0.002
Total CPR time (min) [a]	0.227	0.039	0.090
Total epinephrine dose (mg) [a]	0.247	0.026	0.003
LDH (U/L), reference value (<290 U/L) [a]	0.054	0.064	0.013

[a] Eta squared. [b] Cramer's v.

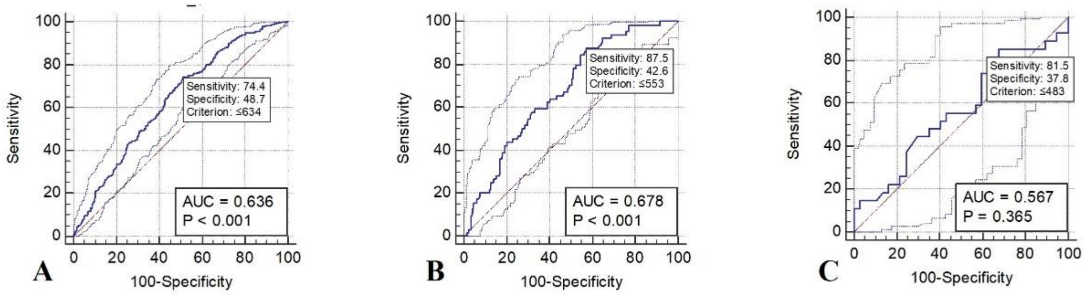

Figure S1. (**A**) Primary outcome (ROSC). (**B**) Secondary outcome (Survival discharge). (**C**) Tertiary outcome (Favorable neurologic outcome).
